# OxyVita^®^C Hemoglobin-Based Oxygen Carrier Improves Viability and Reduces Tubular Necrosis in Ex Vivo Preserved Rabbit Kidneys

**DOI:** 10.3390/ijms26199266

**Published:** 2025-09-23

**Authors:** Waldemar Grzegorzewski, Łukasz Smyk, Łukasz Puchała, Leszek Adadynski, Marta Szadurska-Noga, Joanna Wojtkiewicz, Maria Derkaczew, Jacek Wollocko, Brian Wollocko, Hanna Wollocko

**Affiliations:** 1 Faculty of Biology, Nature Protection and Sustainable Development, University of Rzeszow, str. Zelwerowicz 4, 35-601 Rzeszow, Poland; 2 Department of Pharmacology and Toxicology, Faculty of Medicine, University of Warmia and Mazury in Olsztyn, Al. Warszawska 30, 10-082 Olsztyn, Poland; lukasz.smyk@uwm.edu.pl (Ł.S.); lukasz.puchala@uwm.edu.pl (Ł.P.); 3 Polish Agency for Health Technology Assessment and Tariff System (AOTMIT), 00-032 Warszawa, Poland; 4 Department of Transplantology and General Surgery, Provincial Specialist Hospital in Olsztyn, str. Zolnierska 18, 10-561 Olsztyn, Poland; adadleszek@o2.pl; 5 Department of Pathomorphology, Faculty of Medicine, Collegium Medicum, University of Warmia and Mazury in Olsztyn, Al. Warszawska 30, 10-082 Olsztyn, Poland; marta.szadurska@uwm.edu.pl; 6 Department of Physiology and Pathophysiology, Faculty of Medicine, Collegium Medicum, University of Warmia and Mazury, Al. Warszawska 30, 10-082 Olsztyn, Poland; mariaderkaczew@gmail.com; 7 OXYVITA Inc., Middletown, NY 10940, USA; jacekw@oxyvita.us (J.W.); hannaw@oxyvita.us (H.W.); 8 Department of Ophthalmology, SUNY Downstate Health Sciences University, Brooklyn, NY 11203, USA; bwollock@gmail.com; 9 Department of Basic Sciences, Touro College of Osteopathic Medicine (TouroCOM), Middletown, NY 03577, USA

**Keywords:** hemoglobin-based oxygen carrier (HBOC), OxyVita^®^C, ex vivo kidney preservation, tubular necrosis, kidney transplantation, aerobic metabolism, oxidative stress, oxygen delivery, Krebs-Ringer’s solution, high-molecular-weight polymer

## Abstract

Organ transplantation has significantly progressed since the 1950s, with notable advancements in surgical procedures and immunosuppression. However, current organ preservation techniques, mainly static cold storage, have not evolved at the same pace and remain insufficient to prevent ischemic and oxidative damage. This damage, primarily caused by the cessation of aerobic metabolism, limits organ viability and transplant outcomes. In this study, we investigated whether supplementing a storage solution with a hemoglobin-based oxygen carrier (HBOC) could improve the condition of ex vivo rabbit kidneys by maintaining oxygenation and supporting aerobic metabolism. In a paired, randomized design, contralateral rabbit kidneys were preserved either in a Krebs-Ringer-based solution enriched with the polymerized hemoglobin OxyVita^®^C (15 g/L, p50 4–6 mmHg, MW ≈ 17 MDa, pH adjusted to 7.4) or in an HBOC-free control solution. Physicochemical characterization of OxyVita^®^C included oxygen equilibrium curves, zeta potential, polydispersity index, and dynamic light scattering. Biochemical markers (AST, ALT, LDH, K^+^, pH) and histopathological assessments were used to evaluate tissue integrity over 24 h. Histology was additionally stratified according to rinsing protocols (unwashed, NaCl single flush, triple flush), and tubular necrosis was scored by blinded pathologists. Group comparisons were analyzed using ANOVA with Tukey’s HSD test. The HBOC-enriched solution showed improved tissue preservation, higher cell survivability, and better histomorphological profiles, with significantly reduced tubular necrosis scores compared to controls. These findings suggest that active oxygen delivery via HBOCs offers a promising strategy to mitigate ischemic damage during ex vivo kidney storage. Limitations include the lack of transplantation outcomes and direct ROS quantification, which will be addressed in future work integrating hypothermic and normothermic machine perfusion.

## 1. Introduction

The process of organ transplantation is complex and features a multitude of elements to maintain organ viability. Despite significant improvements in surgical techniques, aseptic processes, and immunosuppressive treatments in recent years, the main cause of failure in this field still lies in the condition of the transplanted organ itself [1,2].

Almost immediately after a transplanted organ is removed from the body, the deterioration process begins. Changes to the organ’s environment, due to ischemia and hypothermia, lead to a decrease and subsequent cessation of normal aerobic metabolism, resulting in anaerobic metabolism. These changes cause damage at the cellular level, affecting both intercellular and intracellular structures [3,4]. The degree of these changes is directly related to the duration of storage and transportation between the donor and the recipient, reflecting the length of time the organ is deprived of oxygen and aerobic metabolism. Thus, the total time that an organ endures ex vivo throughout the transplantation process affects its pathomorphology and, consequently, the success of the surgery and the patient’s recovery [5,6].

Several FDA-approved preservation solutions are available, with more under active modification or development, aimed at maintaining the functional condition of the transplanted organ during transportation [7]. However, their ability to maintain full cellular viability and prevent ischemia–reperfusion injury remains limited [7,8]. Their compositions are designed to preserve the organ by supplying sugars, ions, and sometimes anti-inflammatory or antimicrobial components [7]. In conjunction with reduced temperatures, these solutions can slow adverse changes but cannot fully prevent the shift from aerobic to anaerobic metabolism, which eventually leads to cellular dysfunction and death [8]. Active oxygen delivery remains a major limitation in conventional preservation strategies [9]. Instead, they rely on oxygen delivered as a “pillow” on top of the preservation solution in perfusion instruments [7].

Since oxygen has limited solubility in water (5.09 mL/L at 25 °C), this method of oxygen delivery to harvested tissue cannot effectively support the metabolic activities of an ex vivo organ. Additionally, excessive oxygen beyond solubility limits can lead to free oxygen radical formation near the organ, potentially causing oxidative stress. Without the circulatory system to neutralize these radicals, accumulation can result in membrane damage, ion imbalance, osmolarity changes, acidosis, and ultimately cell death [10].

This article aims to assess whether adding an active oxygen carrier to preservation solutions can improve the condition of an ex vivo organ. Hemoglobin-based oxygen carrier (HBOC) therapeutics can provide effective oxygen delivery within the organ’s microcirculation [11]. In ex vivo settings, the HBOC dissolved in the preservation solution allows active oxygen diffusion directly to organ tissues. This approach could help maintain metabolism and stabilize organ conditions, potentially increasing the survival of organ tissues and cells during transplantation.

Two solutions containing metabolites and sugar were used. One solution, O2A-005, contained an oxygen carrier, while the other was Krebs-Ringer’s solution, previously used in organ storage [12,13]. The composition of both solutions differs from many other preservation solutions, which usually contain additional sugars, amino acids, antioxidants, or colloids [7]. The study, therefore, evaluates the specific contribution of the active oxygen carrier (OxyVita^®^C) to organ preservation, independent of other additives. The O2A-005 solution was engineered with a nano-polymer form of hemoglobin, designed to mimic blood’s oxygen delivery function and allow continued aerobic metabolism in the ex vivo organ.

The HBOC used, OxyVita^®^C, is a next-generation oxygen carrier with a polymer size that prevents extravasation and reduces risks of hypertension and renal toxicity associated with earlier HBOCs [14,15]. Its low p50 (4–6 mmHg) allows oxygen release preferentially within hypoxic microcirculation, minimizing over-oxygenation and oxidative radical formation [14,15]. The HBOC in this study was provided as a powder with lower than usual Hb concentration (1.5 g%), optimized for dissolution in O2A-005 and effective oxygen transport.

The extent of cell and tissue damage in rabbit kidneys was monitored using AST, ALT, LDH, potassium, and pH, as well as histopathology after 24 h. Samples of perfusion solutions were collected at 6, 12, and 24 h. Baseline measurements (“time zero”) were established using healthy rabbit blood for reference.

It is beyond the scope of this publication to compare different commercially available solutions. This study focuses on evaluating whether an active oxygen carrier mechanism improves ex vivo organ condition. Selected enzymes (LDH, AST, ALT) are normally present at low concentrations in blood but rise during cell damage, making them reliable markers for tissue deterioration [16].

## 2. Results

The study compared ex vivo kidney storage under anaerobic conditions using Krebs-Ringer’s solution as a control and the investigational O2A-005 solution intended to provide a more aerobic environment. (The compositions of the solutions are presented in Table 1A,B.)

### 2.1. Biochemical Analysis

**Figure 1:**
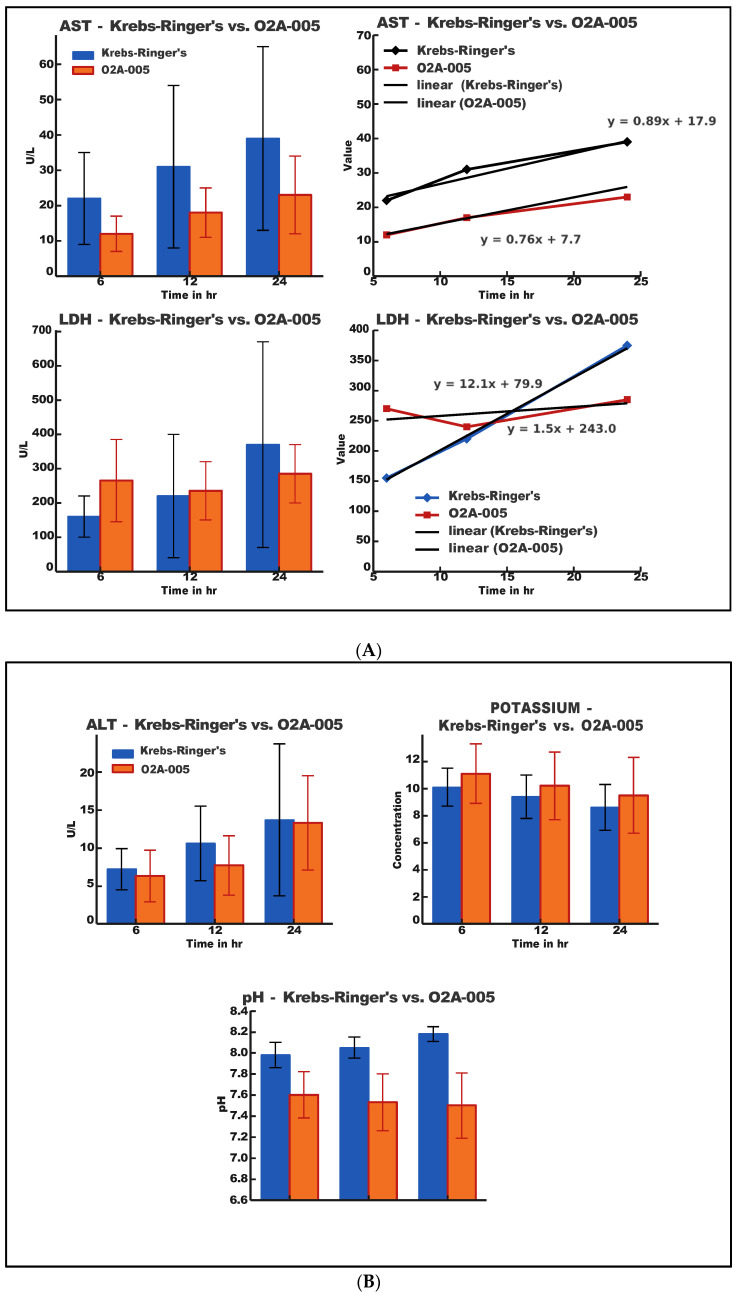
(**A**)**.** Results of analysis of aspartate aminotransferase (AST), limit of 10 to 40 units; and lactic acid dehydrogenase (LDH), limit of 100–190 unit/liter in organs stored in the O2A-005 compared to the Krebs-Ringer’s solution. Error bars are included. (**B**)**.** Results of analysis of alanine aminotransferase (ALT), limit of 7 to 56 units, potassium ion concentration, limit of 3.5 to 5.0 mEq/L, and pH changes in organs stored with the O2A-005 compared to the Krebs-Ringer’s Solution. Error bars are included.

The tissue damage in stored kidneys was assessed by measuring AST, ALT, LDH, potassium, and pH at 6, 12, and 24 h. “Time zero” values were based on healthy rabbit serum. Results are summarized in Figure 1A,B and Table 2.

**Figure 2:**
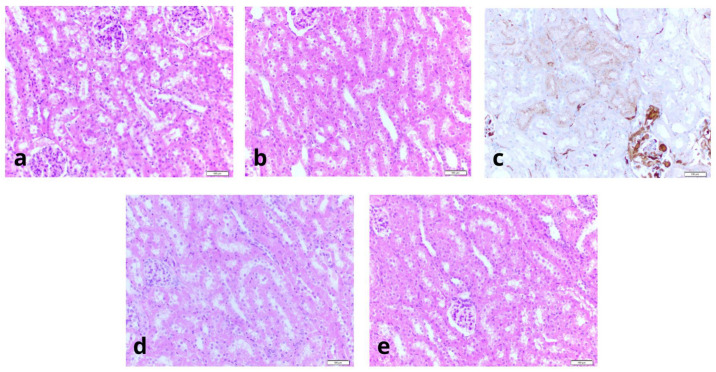
(**a**). The level of tubular necrosis in Oxyvita samples has been assessed to be between 85% and 100%, and the type of solution, that was used to rinse kidney, seemed to affect the level of necrosis. All, or almost all, features of the acute tubular necrosis (ATN) have been found in the evaluated material: cytoplasm swelling and vacuolation, flattening and shedding of epithelial cells, eosinophilic casts and debris within lumina of tubules; (**b**). ATN observed in kidneys rinsed with Oxyvita seemed to be at earlier stage of development; furthermore, the distribution of changes was uneven and it showed a pattern that could be described as “segmental”. ATN in kidney rinsed with Ringer solution was advanced and uniformly distributed.; (**c**). Large casts of hemoglobin within glomerulus including interior of the Bowman capsule and dispersed hemoglobin present within cytoplasm of tubular epithelia. (**d**). Non-rinsed, “0”-control kidney. Massive and widespread ATN hallmarks observed. (**e**). Ringer solution rinsed kidney. We have found canulation of kidney vessels in order to rinse organ as the best method to minimalize level and extent of acute tubular necrosis. Bar scale 100 µm.

**Figure 3:**
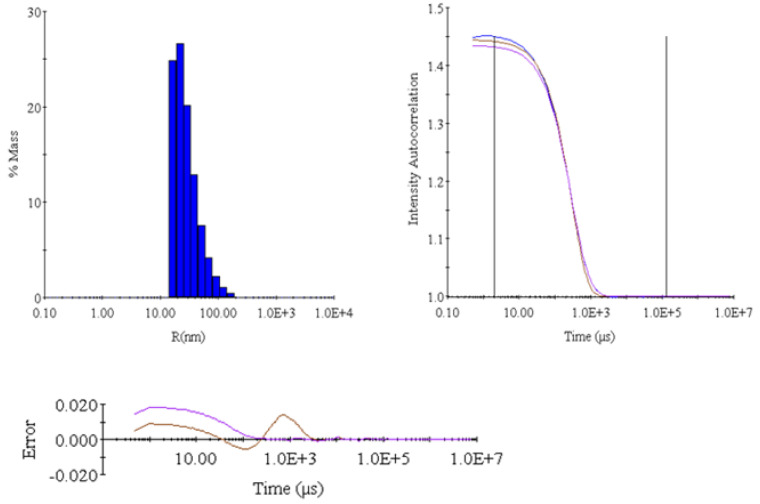
Dynamic light scattering (DLS) analysis of the OxyVita^®^C product, including molecular weight, radius, and uniformity of the polymer.

Table 2, Table 3 and Table 4 contain a statistical analysis of the obtained data. Additionally, the organ’s damage evaluation was assessed by histopathological examination of the tissue. Figure 2 shows the histopathology findings. Table 5 and Figure 3 show the results of DLS analyses, given as a reference for the size and uniformity of the OxyVita^®^C.

**AST:**

AST levels increased over time in both solutions but were substantially lower in the O2A-005 group. Linear regression analysis showed a slope of m = 0.89 for Krebs-Ringer’s solution (y = 0.89x + 17.9) and m = 0.76 for O2A-005 (y = 0.76x + 7.7). Total AST increase over 24 h was 38.8 U/L in the control vs. 25.7 U/L in O2A-005, indicating reduced cellular damage in the presence of the HBOC.

**LDH:**

LDH levels were markedly elevated in the Krebs-Ringer’s solution group compared to O2A-005, particularly within the first 6 h (158 vs. 265 U/L). Slopes from linear regression were m = 12.1 (control) and m = 1.5 (O2A-005), indicating a slower rate of cellular injury in the O2A-005 group. LDH increases were interpreted against normal serum ranges (100–190 U/L).

**ALT:**

ALT levels remained relatively low throughout storage, with slightly lower values in the O2A-005 group (24 h: 13.2 U/L) compared to the control (13.6 U/L). The Tukey HSD post hoc test confirmed significant differences at 24 h (*p* = 0.0015).

**Potassium and pH:**

Both solutions maintained buffering capacity. pH values ranged from 7.5 to 7.6 in O2A-005 and 8.0–8.2 in Krebs-Ringer’s solution. Potassium levels slightly decreased over time in both groups, with a trend towards lower accumulation in O2A-005.

**Statistical analyses:**

ANOVA (Fisher test, Bonferroni correction) indicated significant differences for AST and ALT over time (*p* < 0.01) and a trend for potassium (*p* = 0.092). Other parameters did not reach significance, likely due to small sample size (Table 3 and Table 4). AST and LDH values increased significantly more in the Krebs-Ringer’s group than in the O2A-005 group, suggesting that the O2A-005 solution reduced cellular injury (ANOVA *p* < 0.01 for AST).

ALT levels remained significantly lower in the O2A-005 group over time (*p* < 0.01). LDH levels showed a non-significant trend toward reduction (*p* = 0.47).

### 2.2. Histopathology

Figure 2 illustrates representative kidney pathology. Histopathological analysis showed no thrombosis or embolism, no casts in Krebs-Ringer’s samples, and small eosinophilic protein casts in O2A-005 group capillaries (diameter < 0.1 mm). The IHC staining confirmed RBC origin of casts.

Overall, tissue architecture was better preserved in O2A-005 kidneys, supporting the biochemical data indicating reduced cellular injury.

### 2.3. Dynamic Light Scattering (DLS) Analysis

**Figure 4:**
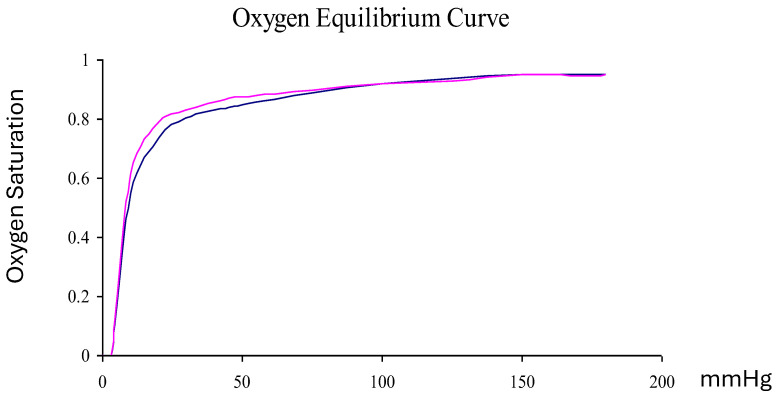
P_50_ of Oxyvita^®^ finished product: solution (Violet) and powder form reconstituted after lyophilization (Blue).

**Figure 5:**
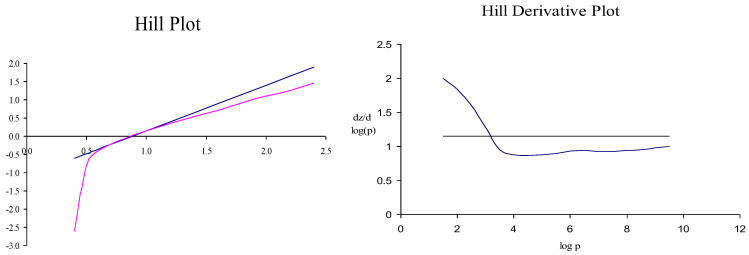
A hill parameter determination for Oxyvita^®^ finished product.

DLS analysis of O2A-005 (Table 5, Figure 3, Figure 4 and Figure 5) confirmed the nano-polymer structure of OxyVita^®^C, uniform in size with an average radius of 39.4 nm, and molecular weight ~17.6 MDa. These data validate the integrity and consistency of the HBOC used for organ preservation. The polydispersity index (PDI) for the polymer is 0.69.

**Table 5:** Dynamic light scattering (DLS) analysis of the O2A-005 solution including size of the polymer, molecular weight, and radius of the nano-polymer.

Item	Intensity (Cnt/s)	R (nm)	%Pd	MW (kDa)
Mean	2,498,440	39.3658	99.5	17,569
S	31,056	0.596	0.687	312.5
%S	1.24	1.514	0.6905	1.7787
S^2^	964,475,136	0.3552	0.4729	97,656

### 2.4. Summary of Key Findings

1. O2A-005 solution slowed the accumulation of AST and LDH compared to Krebs-Ringer’s solution.
2. ALT release was minimal in both groups but slightly lower in O2A-005.
3. pH and potassium remained within physiological ranges; O2A-005 maintained slightly more stable values.
4. Histopathology showed minimal tissue damage in O2A-005-stored kidneys, with RBC-derived capillary casts only in O2A-005.
5. DLS confirmed polymer stability and uniformity in O2A-005.
6. The presence of OxyVita^®^C in the storage solution created a more aerobic environment, preserving cellular integrity and reducing biochemical and histological markers of damage during ex vivo kidney storage.

## 3. Discussion

The zero-link technology used in the preparation of the oxygen carrier enables the production of a nano-polymer with remarkable strength and uniform size distribution. This process eliminates potentially toxic residual chemicals, ensuring that when the polymer is metabolized, no harmful by-products are released. Moreover, the higher molecular weight of this nano-polymer compared to earlier products helps prevent extravasation, while its oncotic pressure and viscosity closely resemble those of plasma [17,18].

When considering the addition of an oxygen carrier to a preservation solution, cellular and tissue metabolism must be taken into account. A critical event after organ harvesting is the shift from aerobic metabolism to anaerobic glycolysis [19]. Immediately after cessation of blood flow and oxygen delivery, numerous changes occur, including alterations in membrane lipid composition, ionic balance, enzyme activity, pH, osmolarity, and temperature [20,21]. Rapid disruptions in sodium and potassium gradients, primarily due to ATPase pump failure, are among the key contributors to metabolic collapse [22]. Declining ATP production and lactic acid accumulation further decrease cellular buffering capacity, impair mitochondrial function, and accelerate cell death [23].

Oxidative stress is another major contributor to post-harvest injury. Reactive oxygen species (ROS), such as superoxide radicals, cause cellular and tissue damage if not neutralized effectively [24,25]. At low levels, ROS may activate survival mechanisms, but prolonged imbalance promotes apoptosis [26]. Supporting oxygen delivery during storage can help maintain aerobic metabolism, thereby reducing harmful metabolite buildup and slowing tissue deterioration.

In this study, levels of ALT, AST, LDH, potassium, and solution pH were assessed over 24 h of kidney storage in O2A-005 or Krebs-Ringer’s solution. LDH was particularly informative, as it is released upon cellular injury and reflects metabolic stress. Organs stored in Krebs-Ringer’s solution showed nearly double the LDH concentration within the first six hours compared to O2A-005. Linear regression confirmed a much steeper increase for the Krebs-Ringer’s group (m = 12.1) versus O2A-005 (m = 1.5), suggesting that O2A-005 slows the rate of cellular injury. LDH levels in O2A-005 remained closer to physiological reference values (100–190 U/L), highlighting its potential to mitigate tissue damage.

The biochemical role of LDH further supports this finding. While it allows glycolysis to continue under anaerobic conditions, it simultaneously drives lactic acid accumulation. Without circulation to clear metabolites, lactic acidosis worsens, amplifying cellular stress. By maintaining aerobic metabolism, O2A-005 helps prevent this cascade. Importantly, oxygen carriers with low p50 values and high oxygen affinity, such as OxyVita Hb, may be particularly suitable for storage solutions, as they prevent tissue over-oxygenation and minimize ROS formation [27,28,29].

ALT and AST, though primarily associated with liver injury, are also useful indicators of renal cell damage [30,31,32]. In this study, both enzymes were consistently lower in kidneys stored with O2A-005. AST accumulation rates were slower in O2A-005 (m = 0.76) compared with Krebs-Ringer’s solution (m = 0.89), again indicating reduced injury. ALT values remained close in both groups but trended lower with O2A-005. Together, these markers reinforce the benefit of supporting aerobic conditions during storage.

Potassium levels did not differ significantly between groups, but monitoring remains clinically relevant, as abnormal potassium concentrations can impair cardiac and muscular function [33,34]. Regarding pH, both solutions maintained buffering capacity throughout 24 h, yet O2A-005 preserved values closer to physiological levels (7.5–7.6 vs. 8.0–8.2 for Krebs-Ringer’s), potentially favoring cellular homeostasis [35].

Histopathological analysis further supported the biochemical findings. Organs stored in O2A-005 exhibited 15–20% less tubular necrosis than those in Krebs-Ringer’s solution. No thrombosis or thromboembolism was observed in either group. Small eosinophilic proteinaceous casts were detected in O2A-005 samples, but follow-up rinsing experiments indicated that these casts likely originated from residual red blood cell fragments rather than intrinsic solution toxicity. Overall, kidneys preserved in O2A-005 demonstrated improved tissue integrity.

Taken together, these results suggest that O2A-005 supports aerobic metabolism, reduces cellular injury markers, and improves histopathological outcomes compared to Krebs-Ringer’s solution. The main limitations of this study are the relatively small sample size and the restricted biomarker panel, which reduce statistical power and may have masked some differences. In addition, functional transplantation outcomes, including delayed graft function and reperfusion injury, were not assessed. Future studies should therefore evaluate HBOC-based solutions in larger experimental cohorts, incorporate broader biomarker panels, and extend analysis to transplantation models to confirm clinical relevance.

## 4. Materials and Methods

### 4.1. The O2A-005 Storage Solution

The O2A-005 storage solution was prepared with the oxygen carrier, OxyVita Hb. This solution was used as a storage and perfusion medium for ex vivo organs. The solution was prepared by dissolving OxyVita^®^C (powder form) in Krebs-Ringer’s solution, with a final hemoglobin concentration of 1.5 g/dL.

This study represents the first experimental use of a powdered HBOC as a component of an ex vivo organ storage solution. The composition of both solutions used in this study is presented in Table 1A,B.

The OxyVita Hb used is a zero-linked polymer of purified bovine hemoglobin, available as a liquid (OxyVita™) or powder (OxyVita^®^C). The current study used the powder form, OxyVita^®^C.

Bovine hemoglobin was chosen as the raw material due to its widespread availability. Fresh blood was obtained from USDA-approved slaughterhouses, and hemoglobin was extracted by lysing red blood cells with a low ionic strength phosphate buffer (pH 7.4). Cellular debris was removed by centrifugation at 3000–4000 RPM. Purified tetrameric hemoglobin underwent β-β cross-linking with [bis(3,5-dibromosalicyl-adipate)] to increase stability and prevent dimerization.

Tetrameric hemoglobin then underwent zero-linked polymerization using chemical activators. No residual chemicals remain in the final polymer. The carbodiimide EDC [1-ethyl-3-(3-dimethylaminopropyl) carbodiimide] activates carboxylate groups on Glu and Asp side chains, forming stable amide (pseudo-peptide) bonds with lysyl residues of adjacent tetramers. To improve reaction efficiency, sulfo-NHS was added, forming intermediate esters that increase polymerization yield.

The powder form (OxyVita^®^C) was obtained by lyophilization or spray-drying of the liquid form. The molecule was pretreated with a protective sugar solution. Reconstitution time was 10–30 s.

### 4.2. Methodology

The New Zealand white rabbit was chosen for its well-characterized physiology, immunology, and ease of handling. A validated rabbit kidney transplant model was used [36].

### 4.3. Animal Husbandry

Experiments followed pre-approved protocols minimizing animal suffering. Procedures adhered to the Polish Ministry of Science and Higher Education guidelines and the NIH Guide for the Care and Use of Laboratory Animals (NIH Publications No. 8023, revised 1978). The study was approved by the Local Ethics Committee, University of Warmia and Mazury in Olsztyn (decision No. 2/N/2004 and No. 32/N/2002).

Twenty female rabbits (4.0–5.8 kg) were acclimated and fed standard chow and water ad libitum until 24 h before the experiment.

### 4.4. Surgical Procedure and Protocol Design

Rabbits were anesthetized locally with 1% lidocaine ointment. Heparin (600 μL) was injected via the lateral auricular vein. General anesthesia was induced using 40 mg/kg ketamine IM, with maintenance IV ketamine–saline (1:1) every 15 min until loss of eyelid reflex.

Aortic cannulation was performed (0.8 mm catheter), and the abdominal aorta and inferior vena cava were clamped above the left renal artery. Warm ischemia start time was recorded. The inferior vena cava was cut, and cold Ringer’s solution (~7 °C) was perfused via the cannulated aorta.

Rabbits were euthanized with 150–300 mg pentobarbital. Cold Krebs-Ringer’s solution (4 °C) was introduced to the lumbar fossa; kidneys were excised with abdominal vessels, maintaining continuous rinsing.

The renal artery was cannulated (0.7–0.8 mm Fi catheter) and rinsed with 10–15 mL cold solution (Krebs-Ringer’s or O2A-005). Samples from the renal vein (5 mL) were frozen at −44 °C for later analysis. Cold ischemia start time was recorded.

Kidneys were stored in solution at 4 °C. At 6, 12, and 24 h, 5 mL solution was perfused, samples collected and frozen. Temperature and pH were recorded at each time point. Kidney tissue (1 cm) was fixed in 4% formaldehyde for macro- and microscopic evaluation.

### 4.5. Experimental Design/Protocol Summary

Each rabbit yielded two kidneys. **Group 1** (n = 8) was flushed with Krebs-Ringer’s solution; **Group 2** (n = 12) was flushed with O2A-005. Kidneys were stored at 4 °C in the respective solutions for 24 h.

### 4.6. Analytical Data

Analyses included potassium, pH, LDH, AST, and ALT at all time points. Tests were performed at a contract laboratory, Olsztyn, Poland. O2A-005 characterization was performed at OXYVITA Inc., New Windsor, NY, USA.

### 4.7. Analytical Methods

#### 4.7.1. UV/Vis Method

Concentration of hemoglobin (g/dL) was determined using UV/Vis spectrophotometry (Hewlett Packard 8452A, Palo Alto, CA, USA) at 538 nm with baseline correction at 680 nm. Samples were diluted 1:100 in 50 mM phosphate buffer (pH 8.0) saturated with CO.

Formula:

g/dL Hb = (Abs538 − Abs680) × 100/0.837/10(1)

Oxyhemoglobin ratio (576/542 nm) was measured with O_2_-saturated buffer. Acceptable ratio = 0.98 ± 0.04.

MetHb content = g/dL Hb with dithionite − g/dL Hb without dithionite.(2)

Results: OxyVita^®^C: Hb = 1.5 g/dL; ratio = 0.986; MetHb = 0.015 g/dL.

#### 4.7.2. Size Exclusion Chromatography (SEC)

SEC was used to verify polymer uniformity. Column: 10 mm × 600 mm packed with Fractogel; mobile phase: 20 mM sodium phosphate + 17.53 g/L NaCl; flow: 0.7 mL/min. Molecular weight of OxyVita^®^C = 17 MDa.

#### 4.7.3. Dynamic Light Scattering (DLS)

DLS measured size distribution and diameter using a DynaPro-801 system. Results are presented in Table 4 and Figure 3.

#### 4.7.4. Osmotic Pressure

Measured with Advanced^®^ Osmometer 3D3S on 0.25 mL samples. Results are provided under Table 1B.

#### 4.7.5. pH Determination

Measured with calibrated Fisher 25 pH-meter (pH 4–10). Initial values are under Table 1B.

#### 4.7.6. p50 Determination

The measurement was performed using Hemox Analyzer with HAS-200 Module. The resulting oxygen dissociation carve is presented in Figure 4.

#### 4.7.7. LDH, AST, ALT Determination

Measured using Cormay Accent 200 analyzer (340/450 nm). Correction factor for Hb absorbance was applied based on matched-concentration spectra. Results: Table 2, Figure 1A,B.

#### 4.7.8. Potassium Determination

Measured with Rapidlab 348 analyzer (Siemens) using pH 6.8/7.3 buffers. Results: Table 2, Figure 1B.

### 4.8. Pathology

Kidneys (n = 20) were fixed in 4% buffered formalin, paraffin-embedded, and stained with H&E. One sample was additionally analyzed via IHC for hemoglobin. Tissue was collected from cortex, medulla, capsule, and perirenal adipose tissue.

Samples were rinsed with NaCl (18 well-rinsed, 2 exceptionally well-rinsed).

Necrosis was quantified using the formula:

M/1000 × 100%(3)

where M = number of necrotic tubules out of 1000 evaluated.

### 4.9. Statistical Analysis

Two-way ANOVA was used to assess group × time differences. Normality was checked via residual histograms; Levene’s test evaluated variance homogeneity. Tukey’s HSD test was used for post hoc comparisons.

Linear regression was applied per group to evaluate time-dependent parameter changes. Slopes of LDH and AST lines were compared.

All statistical analyses were conducted by Lech Zareba, PhD (University of Rzeszów, Poland) using R v4.3.1.

## 5. Conclusions

This study demonstrates that supporting aerobic metabolism during ex vivo kidney storage improves tissue integrity and reduces cellular injury compared to conventional anaerobic solutions. The O2A-005 solution, due to its oxygen-carrying capacity, limited acute tubular necrosis and slowed the accumulation of injury markers. These findings suggest that hemoglobin-based oxygen carriers (HBOCs) may extend the preservation window and help maintain organs closer to physiological normality prior to transplantation. While our study was limited to ex vivo storage, further research is needed to evaluate HBOCs in perfusion systems and transplantation models, with a focus on graft function and long-term outcomes.

## Figures and Tables

**Table 1:** (**A**)**.** Composition of Krebs-Ringer’s solution. (**B**). Composition of O2A-005 solution.

(**A**)					
**Krebs-Ringer’s Composition**		**Molecular Formula**	**MW**	**Krebs-Ringer’s Solution Composition (1000 mL)**	
			**g/mol**	**g**	**mM**
1	Sodium Chloride,	NaCl	58.4	7.02	120.2
2	Sodium Bicarbonate,	NaHCO_3_	84.0	1.302	15.5
3	Magnesium Chloride Hexahydrate,	MgCl_2_·6H_2_O	203.3	0.244	1.2
4	Sodium Phosphate, Monobasic	NaH_2_PO_4_	119.98	0.188	1.3
5	Potassium Chloride,	KCl	74.6	0.44	5.8
6	Calcium Chloride,	CaCl_2_	110.98	0.28	1.9
7	D-glucose (dextrose),	C_6_H_12_O_6_	180.16	2.08	11.6
(**B**)					
**O2A-005 Composition**		**Molecular Formula**	**MW**	**Krebs-Ringer’s Solution composition (1000 mL)**	
			**g/mol**	**g**	**mM**
1	Krebs-Ringer’s	See Table 2			
2	Hb	Polymer	17MDa	15	15

**Table 2:** Results and Statistical Data. Analytical results of ALT, AST, LDH, potassium concentration, and pH changes in samples taken from organs stored using the Krebs-Ringer’s Solution and O2A-005 solution at 6, 12, and 24 h of storage.

**ALT**	**Krebs-Ringer’s**		**O2A-005**	
Time (h)	Mean	SD	Mean	SD
6	7.25	2.605	6.44	3.47
12	10.63	4.809	7.72	3.98
24	13.63	10.084	13.21	6.20
**AST**	**Krebs-Ringer’s**		**O2A-005**	
Time (h)	Mean	SD	Mean	SD
6	22.00	4.683	11.78	6.44
12	30.63	6.842	17.52	7.72
24	38.75	11.463	25.74	13.21
**LDH**	**Krebs-Ringer’s**		**O2A-005**	
Time (h)	Mean	SD	Mean	SD
6	157.75	61.46	265.32	130.16
12	217.75	179.75	238.49	83.22
24	373.43	270.64	286.57	81.49
**POTASSIUM**	**Krebs-Ringer’s**		**O2A-005**	
Time (h)	Mean	SD	Mean	SD
6	10.18	1.454	11.11	2.125
12	9.44	1.595	10.26	2.495
24	8.51	1.852	9.48	2.844
**pH**	**Krebs-Ringer’s**		**O2A-005**	
Time (h)	Mean	SD	Mean	SD
6	7.98	0.099	7.61	0.248
12	8.05	0.108	7.54	0.275
24	8.16	0.090	7.52	0.316

**Table 3:** Results of analysis of variance; results based on calculations in r version 4.3.1 with ANOVA analysis based on the F (Fisher) test and *p*-Bonfferoni.

Parameter	Factor/Test	Df	Sum Sq/F Value	Mean Sq	F Value	Pr (> F)	Significance
**ALT**	group	1	16.7	16.70	0.521	0.47350	
	time point	2	442.2	221.09	6.898	0.00215	**
	group: time point	2	6.5	3.27	0.102	0.90320	
	Residuals	54	1730.7	32.05			
**AST**	group	1	2084	2084	9.923	0.00266	**
	time point	2	2775	1388	6.608	0.00271	**
	group: time point	2	86	43	0.205	0.81534	
	Residuals	54	11338	210			
**LDH**	group	1	11454	11454	0.529	0.470	
	time point	2	92498	46249	2.136	0.128	
	group: time point	2	65015	32507	1.501	0.232	
	Residuals	54	1169442	21656			
**Potassium**	group	1	14.40	14.40	2.945	0.0920	(trend)
	time point	2	33.89	16.95	3.466	0.0385	*
	group: time point	2	0.59	0.30	0.061	0.9414	
	Residuals	53	259.07	4.89			

Levene’s: test of homogeneity of variance (significance: * *p* < 0.05, ** *p* < 0.01).

**Table 4:** Results of the Tukey HSD-ALT post hoc test (time point).

Comparison	Difference (diff)	Lower Bound (lwr)	Upper Bound (upr)	*p* Value adj	Significance
24–12	3.95	−0.36	8.26	0.079	
6–12	−2.66	−6.97	1.66	0.306	
6–24	−6.61	−10.92	−2.29	0.0015	**

** *p* < 0.01, post hoc: only significant and borderline for ALT.

## Data Availability

The data presented in this study are available on request from the corresponding author.

## Abbreviations

The following abbreviations are used in this manuscript:

ALT	alanine aminotransferase
AST	aspartate aminotransferase
ATN	acute tubular necrosis
DLS	dynamic light scattering
HBOC	hemoglobin-based oxygen carrier
HC	histochemistry
IHC	immunohistochemistry
LDH	lactate dehydrogenase
RBC	red blood cell
